# CRISPR/Cas9-Mediated Gene Disruption Reveals the Importance of Zinc Metabolism for Fitness of the Dimorphic Fungal Pathogen Blastomyces dermatitidis

**DOI:** 10.1128/mBio.00412-18

**Published:** 2018-04-03

**Authors:** Gregory C. Kujoth, Thomas D. Sullivan, Richard Merkhofer, Taek-Jin Lee, Huafeng Wang, Tristan Brandhorst, Marcel Wüthrich, Bruce S. Klein

**Affiliations:** a Department of Pediatrics, University of Wisconsin—Madison, Madison, Wisconsin, USA; b Department of Medical Microbiology & Immunology, University of Wisconsin—Madison, Madison, Wisconsin, USA; c Department of Medicine, University of Wisconsin—Madison, Madison, Wisconsin, USA; Duke University

**Keywords:** CRISPR, fungi, genetics, pathogenesis, virulence

## Abstract

Blastomyces dermatitidis is a human fungal pathogen of the lung that can lead to disseminated disease in healthy and immunocompromised individuals. Genetic analysis of this fungus is hampered by the relative inefficiency of traditional recombination-based gene-targeting approaches. Here, we demonstrate the feasibility of applying CRISPR/Cas9-mediated gene editing to *Blastomyces*, including to simultaneously target multiple genes. We created targeting plasmid vectors expressing Cas9 and either one or two single guide RNAs and introduced these plasmids into *Blastomyces* via *Agrobacterium* gene transfer. We succeeded in disrupting several fungal genes, including *PRA1* and *ZRT1*, which are involved in scavenging and uptake of zinc from the extracellular environment. Single-gene-targeting efficiencies varied by locus (median, 60% across four loci) but were approximately 100-fold greater than traditional methods of *Blastomyces* gene disruption. Simultaneous dual-gene targeting proceeded with efficiencies similar to those of single-gene-targeting frequencies for the respective targets. CRISPR/Cas9 disruption of *PRA1* or *ZRT1* had a variable impact on growth under zinc-limiting conditions, showing reduced growth at early time points in low-passage-number cultures and growth similar to wild-type levels by later passage. Individual impairment of *PRA1* or *ZRT1* resulted in a reduction of the fungal burden in a mouse model of *Blastomyces* infection by a factor of ~1 log (range, up to 3 logs), and combined disruption of both genes had no additional impact on the fungal burden. These results underscore the utility of CRISPR/Cas9 for efficient gene disruption in dimorphic fungi and reveal a role for zinc metabolism in *Blastomyces* fitness *in vivo*.

## IMPORTANCE

*Blastomyces* is a human fungal pathogen that can cause serious, even fatal, lung infections. Genetic analysis of this fungus is possible but inefficient. We applied a recently developed gene editing technology, CRISPR/Cas9, to dramatically improve the efficiency with which gene disruptions are introduced into *Blastomyces*. We used this system to disrupt genes involved in zinc uptake and found that this reduced the fitness of the fungus upon infection.

## INTRODUCTION

Blastomyces dermatitidis is a human fungal pathogen endemic to the waterways of the Upper Midwest of the United States and the Canadian Great Lakes region and can also be found in parts of Central and South America, Africa, and the Middle East. It is the causative agent of blastomycosis, a lung infection that can progress to a serious systemic disease in healthy persons and especially in immunocompromised individuals. *Blastomyces* is a thermally dimorphic fungus that grows as a mold in soil but is triggered by elevated temperature to transition to a single-cell yeast in the lungs of an infected host. In many cases, expansion of yeast in the lungs generates a Th17-dependent immune response, leading to an influx of neutrophils and fungal containment or clearance (1). If the infection is not contained by the immune system, however, *Blastomyces* can spread to the skin, bone, and genitourinary tract and occasionally to the central nervous system (~5 to 10% of cases) (2). Treatment with antifungal azole drugs or amphotericin B is often effective, but in some cases, blastomycosis can become life-threatening.

We have previously examined changes in gene expression during the *Blastomyces* phase transition from mold to yeast in the context of murine lung infection. Among the most highly induced genes during mouse infection were several that are involved in zinc metabolism, including *PRA1* (pH-regulated antigen 1) and the zinc-regulated transporter genes *ZRT1* and *ZRT2* (3). Zinc uptake has been shown to be important for the pathogenesis of another human fungal agent, Candida albicans (4). In the latter system, Pra1 acts as a secreted “zincophore” to bind extracellular zinc that may be sequestered by the host tissues and subsequently interacts with the high-affinity zinc transporter Zrt1 to promote zinc uptake into the yeast cell. Importantly, *PRA1* or *ZRT1* ablation interferes with the ability of C. albicans to infiltrate epithelial cells (4). In light of our gene expression findings, we sought to determine whether *PRA1* and *ZRT1* are similarly essential for *Blastomyces* virulence.

Several methods of genetic manipulation of *Blastomyces* have been developed, including RNA interference and recombination-based gene targeting (5–7). Because homologous recombination occurs at a low frequency in *Blastomyces*, the efficiency of such traditional approaches has been low, leading to the need to screen large numbers of transformants to find the desired mutants. In contrast, CRISPR/Cas9-mediated gene editing has proven to be a simple and efficient system for genetic modification of many model organisms (8). CRISPR/Cas9 gene editing requires a single guide RNA (sgRNA) with complementarity to the target gene sequence that directs the Cas9 nuclease to generate a double-stranded DNA break at the locus of interest. During the cellular process of repairing the break by nonhomologous end joining (NHEJ), small base insertions or deletions occur, generating frameshifted nonsense proteins and loss of gene function.

In this work, we have succeeded in adapting CRISPR/Cas9 editing to this dimorphic fungus to allow efficient gene disruption. We have generated targeting vectors expressing Cas9 and either one or two sgRNAs to test several loci of interest and introduced these plasmids into *Blastomyces* via *Agrobacterium-*mediated gene transfer. We have compared two approaches to simultaneously target dual loci and found that multiplex targeting is feasible in this fungus. Indeed, the efficiencies of targeting of dual loci were similar to those achieved with single-sgRNA vectors, suggesting no negative effects of a dual-sgRNA strategy. We have also examined the functional consequences of disrupting *PRA1* and *ZRT1* and found that these zinc acquisition genes are important for full fitness in a mouse model of infection.

## RESULTS

### CRISPR/Cas9-mediated gene editing in *Blastomyces* is highly efficient.

**FIG 1:**
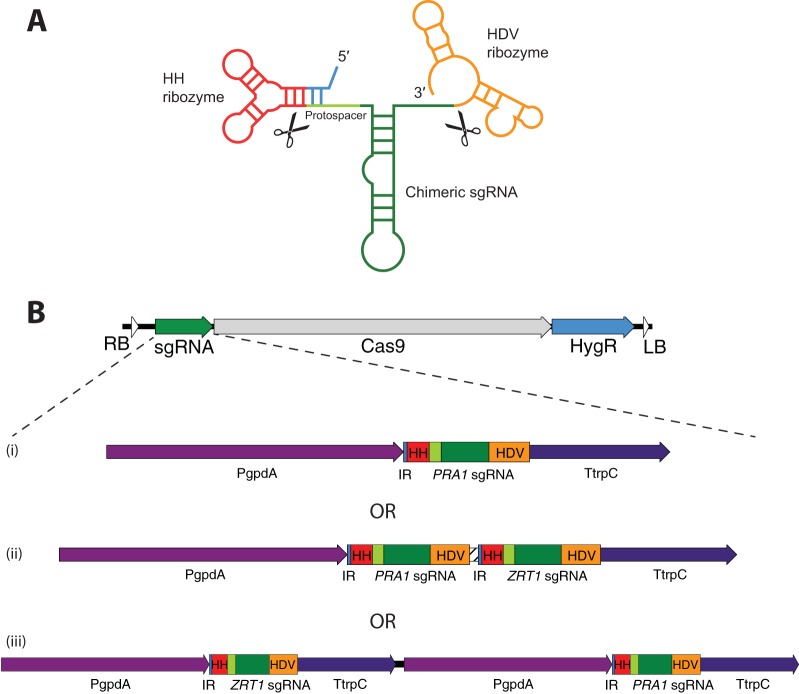
Gene-targeting strategy and vector design. (A) CRISPR-Cas9 gene inactivation is a modification of the approach of Nødvig et al. (9). Primary sgRNA transcripts containing flanking ribozyme sequences are self-excised to generate functional sgRNAs that direct Cas9 cleavage to complementary sites in the genome. Note that the hammerhead (HH) ribozyme forms an inverted repeat with the 5′ end of the target-specific protospacer sequence. (B) Targeting vectors contain one or two sgRNAs to target gene loci, a fungal codon-optimized *Cas9* gene, a hygromycin resistance marker, and border repeat sequences (right border, RB; left border, LB) necessary for *Agrobacterium*-mediated gene transfer into *Blastomyces*. (i) Some constructs contain only single target sgRNA cassettes. (ii) In the single-promoter, dual-targeting constructs, the *PRA1* and *ZRT1* sgRNAs are each embedded within flanking ribozyme sequences, are separated by a linker (hatched bar), and share a single promoter and terminator region driving their expression. (iii) Dual-promoter, dual-targeting constructs have separate promoter-sgRNA-terminator cassettes for each targeted gene. HDV, hepatitis delta virus ribozyme sequence; PgpdA, A. nidulans *gpdA* promoter; TtrpC, A. nidulans *trpC* terminator; IR, inverted-repeat-forming region of the HH ribozyme.

In an attempt to increase the efficiency of gene editing in *Blastomyces*, we employed CRISPR/Cas9 methodology based on the approach of Nødvig et al. (9), which has been successful in targeting filamentous fungi such as *Aspergillus* species. Those investigators constitutively coexpressed in the same plasmid a nuclear localization signal-tagged, fungal codon-optimized Cas9 gene and an RNA polymerase II promoter-driven sgRNA cassette flanked by ribozyme sequences. The primary sgRNA-containing transcript undergoes self-excision of the internal sgRNA by the ribozymes, releasing the active RNA moiety (Fig. 1A). To adapt this system for *Blastomyces*, we modified the Cas9/sgRNA expression vector with the border repeats needed to generate transfer DNA in the *Agrobacterium*-mediated gene transfer system and added a selectable marker of hygromycin B resistance (Fig. 1B). Gene-specific sgRNA cassettes were generated by overlapping PCR fragments and inserted into the vector backbone by using Gibson assembly reactions. The protospacer sequences used for all of the genes we targeted are listed in Table 1.

**TABLE 1:** Efficiency of CRISPR/Cas9 gene targeting in B. dermatitidis

**Target gene**	**Sequence (1–20)**	**PAM**	**Strand**	**% GC**^g^	**Efficiency (%)**	
					**Single target**	**Dual targets**
*BL-ENG2* protospacer 1	AGTACTCAGCTTGGCTAGAG	CGG	Antisense	50	5/6 (83), 7/10 (70)	
*BL-ENG2* protospacer 2	GAAGTCGTCAAAGAAGTTCG	AGG	Antisense	45	3/5 (60) 0/2	
*PRA1* protospacer	GCTGGGTTCCAATCTGCATG	GGG	Antisense	55	8/11 (73), 8/11 (73)	7/9 (78),^a,c^ 21/24 (87),^a,d^ 19/22 (86)^b,e^
*ZRT1* protospacer 1	ATAGCAAGGCAATACGTAGG	GGG	Antisense	52	3/12 (25)	2/9 (22),^a,c^ 1/24 (4),^a,d^ 16/22 (73)^b,e^
*ZRT1* protospacer 2	CATGGTTATGGCAGTCGGAG	AGG	Antisense	57	2/9 (22)	
*mCherry* protospacer v1	GCGCTTCAAGGTGC**G**CATGG^f^	AGG	Sense	65	0/12	
*mCherry* protospacer v2	GCGCTTCAAGGTGCACATGG	AGG	Sense	60	15/24 (63)	
*mCherry* protospacer v3	GCGCATGAACTCCTTGATGA	TGG	Antisense	50	22/30 (73)	

a Dual-promoter, dual-sgRNA strategy.

b Single-promoter, dual-sgRNA strategy.

c *pra1 zrt1* dual-targeted mutants, experiment 1: 2/9 (22%).

d *pra1 zrt1* dual-targeted mutants, experiment 2: 0/24.

e *pra1 zrt1* dual-targeted mutants, experiment 3: 16/22 (73%).

f The boldface base in the protospacer sequence is a mismatch with the target gene.

g %GC excludes the PAM sequence.

We first utilized this system to target *PRA1* of *Blastomyces* (Fig. 1B, diagram i). *Agrobacterium*-mediated transformation of the *PRA1* targeting vector into B. dermatitidis strain 26199 yeast cells yielded 71 transformants, of which 22 were screened by PCR amplification and sequencing of the *PRA1*-targeted region. We observed that 16 clones (73%) displayed insertions or deletions at the expected Cas9 cut site, 3 bases upstream of the protospacer-adjacent motif (PAM) (Table 1). As a control, we also introduced a Cas9-expressing vector without any sgRNA cassette into 26199 cells and sequenced the same *PRA1* region of six transformants; these Cas9-only control clones all had wild-type *PRA1* protospacer sequences, as expected. As is typical with NHEJ repair of CRISPR/Cas9-induced double-strand breaks in other model organisms, we observed insertions and deletions at the expected Cas9 cut site of the *PRA1* gene ranging from 1 to 7 bases in length (Table 2). By far, the most commonly observed mutation was insertion of a single G residue, with the longer indels being represented in single clones each.

**TABLE 2:** Mutations observed in targeted transformants^a^

Mutation^b^	*PRA1*	*ZRT1* no. 1	*ZRT1* no. 2	*mCherry* no. 2	*mCherry* no. 3	*BL-ENG2* no. 1	*BL-ENG2* no. 2
del A	1						
del CAG	1						
del CCCAT	1						
del G	1						
del GC	1						
del GCAG	1						
del GCAGA	1						
del TGGCAGA (mixed peaks)	1						
ins A		8		3	2	4	
ins A (mixed peaks)		13		9		5	
ins A/del A (mixed peaks)				1		3	
ins A/del A (triple peaks)				1			
ins C			2				
ins CA (mixed peaks)				1			
ins G	36						
ins G (mixed peaks)	10						
ins G/del G. (mixed peaks)	1						
ins GCAGA	1						
ins GCCAT	1						
ins T	2				5		1
ins T (mixed peaks)					10		2
ins T + ins A (single mixed base)					2		
ins T + ins A/ins G (single mixed base)					1		
ins T/del CAAGGAGTTCAT					1		
ins T/ins A/del T					1		

a Number of transformants with the mutation indicated at the expected Cas9 cut site of the targeted gene; the protospacer version is shown if multiple sgRNAs were used.

b The mutation sequence of the sense strand of the target gene is shown.

The mutation frequency of *PRA1* produced by CRISPR/Cas9 editing was much higher than the approximately 0.05 to 2% typical of traditional homologous-recombination-based gene editing in *Blastomyces* (for example, see reference 7). We sought to determine whether this high targeting efficiency was locus specific, so we designed sgRNA vectors to target several other *Blastomyces* genes, including (i) the high-affinity zinc transporter *ZRT1*, (ii) the recently identified dectin-2 ligand *Blastomyces* endoglucanase 2 (*BL-ENG2*) (10), and (iii) an exogenously introduced *mCherry* gene in an engineered *Blastomyces* strain (11). For these attempts, we chose multiple sgRNA sequences for each target. The editing efficiencies of these genes varied slightly by locus and experiment but, with one exception, were all at least 1 to 2 orders of magnitude higher than typical traditional frequencies, similar to our results at the *PRA1* locus (Table 1). There was one *mCherry* sgRNA design (*mCherry* protospacer v1) that failed to give any mutant clones, and in this case, the protospacer sequence had a single mismatch with the target gene located 6 bases upstream of the PAM. Mismatches near the 3′ end of the protospacer are less well tolerated by Cas9 than those toward the 5′ end, so the lack of targeting in this case is not surprising (12–14). The spectrum of mutations observed at these other targeted genes also consisted primarily of single base insertions and deletions, although a CA insertion and a 12-base deletion were present in one *mCherry* clone each (Table 2).

With such high mutation frequencies being observed with this CRISPR/Cas9 system, we sought to determine whether simultaneous gene editing of multiple targets would be feasible in *Blastomyces*, similar to multiplex applications of CRISPR/Cas9 in other, more tractable, model organisms. To this end, we employed two strategies to express dual sgRNAs. In one approach, *PRA1* and *ZRT1* (protospacer 1) sgRNA cassettes were separated by a linker sequence and both were expressed under the control of a single promoter and transcriptional terminator (Fig. 1B, diagram ii). In the alternate approach, each sgRNA was expressed within separate promoter and transcriptional terminator cassettes (Fig. 1B, diagram iii). The dual-promoter strategy was attempted in two experiments and generated only two clones with indels in both the *PRA1* and *ZRT1* genes, whereas a lone experiment using the single-promoter approach resulted in 16/22 clones (73%) containing mutations at both targeted loci (Table 1). Targeting efficiencies at each individual locus using either dual-targeting strategy were broadly similar to those observed in the single gene-targeting experiments, suggesting that multiplexing does not incur a notable penalty in editing efficiency.

In most CRISPR/Cas9 targeting experiments, we observed a variable proportion of the edited clones displaying multiple sequence peaks beginning at the expected Cas9 cleavage site within the targeted protospacer region. This ranged from 0 to 80% with a median of 56% among all targeting experiments. As *Blastomyces* is a multinucleate fungus, we interpret this to be evidence of heterokaryosis. In some cases, deconvolution of the multiple sequence peaks identified both wild-type and mutant sequences present within a transformant; in other clones, two or more mutations could be discerned in the absence of the wild-type sequence. Extended passage of such “mixed-peak” heterokaryotic mutant clones under continued hygromycin B selection did not result in pure homokaryotic clones (data not shown; see also Fig. S1 in the supplemental material). It should be noted that only homokaryotic clones were used for subsequent experiments assessing gene expression, growth, and *in vivo* fitness. The strains used for such phenotyping are shown in Table S1.

10.1128/mBio.00412-18.1 FIG S1  Extended selection does not drive heterokaryotic clones to homokaryosis. Chromatograms from sequencing of the targeted region of the *mCherry* gene in clones M323 and M330 after 1, 4, or 7 additional passages under hygromycin B selection. The shaded vertical bar indicates the expected Cas9 cut site between 3 and 4 bases upstream of the PAM. Note the presence of clean sequence peaks that transition to multiple peaks under peaks characteristic of multiple indel mutations within a given clone. The two clones shown are from targeting with *mCherry* protospacer version 3, and their multiple specific mutations determined by manual deconvolution are indicated. Download FIG S1, EPS file, 17.6 MB. Copyright © 2018 Kujoth et al. 2018 Kujoth et al. This content is distributed under the terms of the Creative Commons Attribution 4.0 International license.

10.1128/mBio.00412-18.5 TABLE S1  B. dermatitidis mutant strains used for phenotyping in this study. Download TABLE S1, DOCX file, 0.1 MB. Copyright © 2018 Kujoth et al. 2018 Kujoth et al. This content is distributed under the terms of the Creative Commons Attribution 4.0 International license.

### Targeted mutants have impaired *PRA1* mRNA and protein expression.

Nearly all of the insertions and deletions generated in these CRISPR/Cas9 targeting experiments would be predicted to generate frameshifts with consequent premature termination codons in their respective genes’ open reading frames. The two exceptions were in-frame deletions in one *PRA1* (delCAG) transformant and one *mCherry* protospacer v3 (delCAAGGAGTTCAT) transformant that would result in one or four amino acid deletions, respectively. In most organisms, transcripts bearing premature termination codons are often, although not universally, targeted for degradation by nonsense-mediated RNA decay mechanisms (15). To determine whether we could detect an effect of CRISPR/Cas9-induced indels on target gene mRNA expression, we performed a real-time quantitative PCR (RT-qPCR) analysis of total RNA harvested from selected wild-type and *pra1 zrt1* mutant clones.

**FIG 2:**
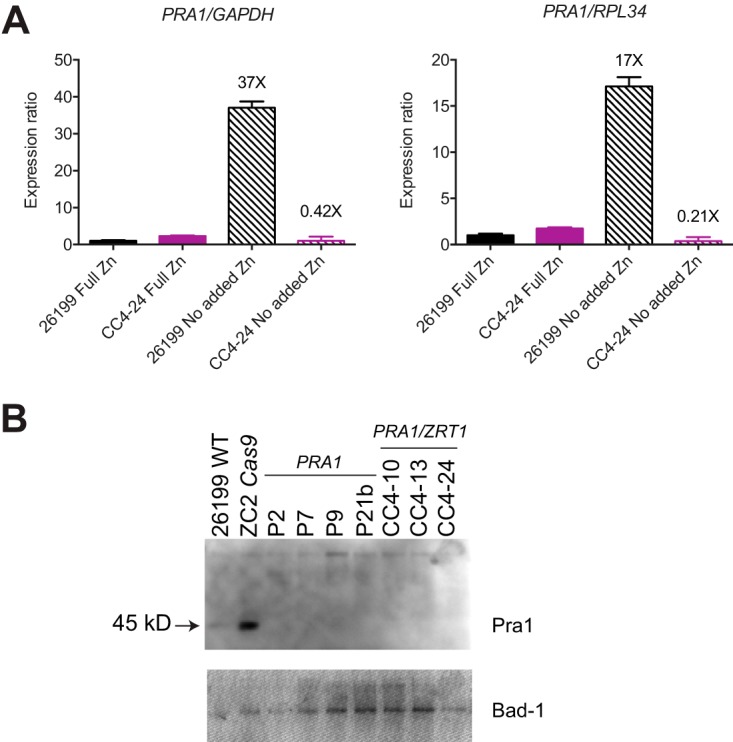
Expression of *PRA1* mRNA and protein is diminished in *pra1* mutant and *pra1 zrt1* double-mutant clones. (A) Total RNA from wild-type 26199 and *pra1 zrt1* dual-targeted mutant clone CC4-24 grown under zinc-replete (Full Zn) and zinc-limiting (No added Zn) conditions was analyzed by RT-qPCR with primers for *PRA1*, *Cas9*, *GAPDH*, and *RPL34*. Expression was normalized to either *GAPDH* or *RPL34* and set relative to 26199 in full Zn. Fold induction values (NAZ/full zinc) calculated by the 2^−ΔΔ*CT*^ method (53) are indicated. Error bars represent standard deviations. (B) Conditioned medium from wild-type (WT) and targeted clone cultures grown in minimal zinc medium were concentrated ~20× prior to Western blot analysis with a polyclonal antibody raised against C. albicans Pra1. The arrow indicates the Pra1 band. Higher-molecular-weight nonspecific bands are present in the lanes and indicate that broadly similar amounts of protein were loaded. The blot was stripped and reprobed with an anti-Bad1 monoclonal antibody (DD5-CB4) to assess the relative protein loading in the lanes (bottom). P2, P7, P9, and P21b are *pra1* mutant clones; CC4-10, CC4-13, and CC4-24 are *pra1 zrt1* double-targeted mutant clones; and ZC2 Cas9 is a control that received only Cas9.

Expression of both *PRA1* and *ZRT1* was found to be very low in the wild-type or *pra1 zrt1* mutant background when cells were grown under zinc-replete conditions (Fig. 2A and data not shown). As expression of *PRA1* and *ZRT1* is inducible by zinc limitation in other fungi (4, 16–18), we examined their transcript levels in cells grown in minimal zinc medium. Under these conditions, *PRA1* transcripts were induced at least 17-fold in wild-type 26199 but showed no induction in the *pra1 zrt1* double-targeted mutant CC4-24 background (Fig. 2A). This is consistent with the expectation of nonsense-mediated mRNA decay of mutated *PRA1* transcripts. Surprisingly, we did not detect robust *ZRT1* mRNA induction under low-zinc conditions in wild-type yeast (2.8-fold), for reasons that are unclear (data not shown).

As expected, we detected Cas9 mRNA expression only in targeted and Cas9-only control clones but not in wild-type 26199 yeast (Fig. S2). This is consistent with the constitutive nature of the *tef1* promoter used to drive Cas9 expression in the targeting vectors (19). Cas9 expression unexpectedly differed under zinc-replete and zinc-limiting conditions, but that should not confound *Blastomyces* fitness *in vitro* or *in vivo*.

10.1128/mBio.00412-18.2 FIG S2  Cas9 expression is constitutive in a targeted clone. Total RNA from wild-type 26199 and *pra1 zrt1* dual-mutant clone CC4-24 grown under zinc-replete (Full Zn) and zinc-limiting (No added Zn) conditions was analyzed by RT-qPCR with primers for Cas9, *GAPDH*, and *RPL34*. Cas9 expression was normalized to either *GAPDH* or *RPL34* and set relative to that of strain 26199 in full Zn. Download FIG S2, EPS file, 1 MB. Copyright © 2018 Kujoth et al. 2018 Kujoth et al. This content is distributed under the terms of the Creative Commons Attribution 4.0 International license.

Although we did not have antibodies raised against *Blastomyces* Pra1 or Zrt1 available for detection of protein expression, we did obtain an antibody against C. albicans Pra1 (20, 21). As the B. dermatitidis and C. albicans Pra1 protein sequences are ~39% identical, we considered that we may be able to detect *Blastomyces* Pra1 protein by using this reagent. Western blot analysis of secreted proteins in *Blastomyces* yeast conditioned medium indeed revealed an expected ~45-kDa band present only in wild-type 26199 and Cas9-only control supernatants from zinc-limited cultures but not in any of the *pra1* or *pra1 zrt1* mutants tested (Fig. 2B). In combination with the qPCR results described above, this supports the finding of impaired zinc-regulated expression of *PRA1* in the targeted clones and indicates that CRISPR/Cas9 editing indeed resulted in loss of protein expression.

### Disruption of *PRA1* or *ZRT1* does not uniformly impair growth in standard medium.

**FIG 3:**
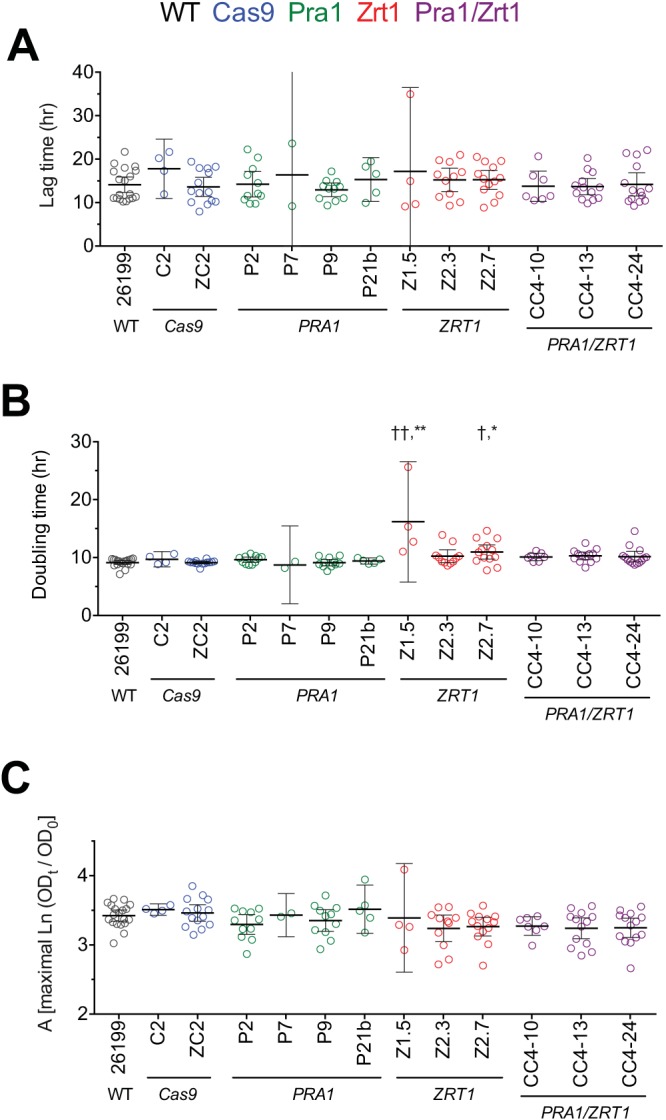
Growth characteristics determined under zinc-replete conditions. The growth of most *pra1*, *zrt1*, and *pra1 zrt1* mutant *Blastomyces* strains in standard liquid HMM is similar to that of parental wild-type (WT) *Blastomyces* strain 26199 or *Cas9*-only (no sgRNA) controls, except for *zrt1* mutant strains Z1.5 and Z2.7. Lag times (A), doubling times (B), and maximal asymptotic growth (C) values were calculated from Gompertz modeling of growth curve data as reparameterized by Zwietering (54). Strains are color coded by targeted gene. Data are pooled from up to eight experiments, although the number of experiments varies by strain. Each value represents an individual growth cycle, and each experiment contained one to four growth cycles. Error bars represent the mean ± the 95% confidence interval. The minimum and maximum 95% confidence intervals of the clipped error bars are 0 and 108 for P7 and 0 and 26.5 for Z1.5. Comparisons with wild-type 26199 (†, *P* < 0.05; ††, *P* < 0.01) and the Cas9-only control (*, *P* < 0.05; **, *P* < 0.01) were made by Kruskal-Wallis analysis with Dunn’s posttest.

We next sought to investigate phenotypic consequences of CRISPR/Cas9 editing in this dimorphic fungus and focused on the two targeted genes involved in zinc metabolism. Prior to embarking upon fitness testing *in vivo*, we characterized CRISPR/Cas9-targeted clones for growth in standard liquid *Histoplasma* minimal medium (HMM) and calculated lag times, doubling times, and maximal growth levels. Multiple independent *pra1* mutant strains showed lag phases, growth rates, and maximal densities similar to those of the wild-type and Cas9-only controls (Fig. 3). Two *zrt1* mutant clones had a variable impact on growth relative to the controls, while others did not (for example, see Z1.5 in Fig. 3). In some experiments, this appeared as an increase in lag time prior to the achievement of growth rates and maximal growth similar to those of controls, whereas in other experiments, the growth rate or maximal growth was somewhat lower than that of the wild type (Fig. 3 and data not shown). The *pra1 zrt1* double mutants displayed occasional variation, as some clones showed increased lag times, decreased growth rates, or decreased maximal growth in individual experiments (but not consistently so), whereas others were similar to wild-type controls. When growth parameters were examined across multiple experiments, the overall variations in growth were within similar ranges for the wild-type, Cas9 control, and *pra1*, *zrt1*, and *pra1 zrt1* mutant strains, except for increased doubling times of *zrt1* mutant strains Z1.5 and Z2.7 (Fig. 3). Thus, we selected mutants with growth similar to that of control strains to test for the potential effect of *PRA1* and/or *ZRT1* loss on fitness *in vivo*.

### *PRA1* and *ZRT1* deficiency reduces the fungal burden in a mouse lung infection model.

**FIG 4:**
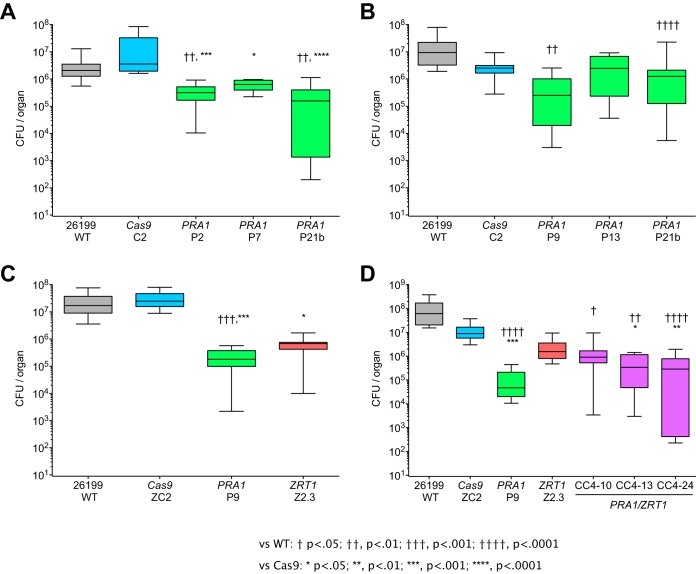
Fitness of wild-type (WT) and mutant *Blastomyces* cells *in vivo*. *pra1* and *zrt1* mutant strains show a reduced fungal burden following intratracheal injection into C57BL/6 mice compared to the wild-type and Cas9-only controls. Approximately 20,000 *Blastomyces* cells were administered per mouse, and lung homogenates were plated for CFU enumeration at 10 to 15 days postinfection. Panels A to D are box plots representing separate experiments with different sets of strains. Comparisons of total numbers of CFU per lung versus wild-type 26199 (†, *P* < 0.05; ††, *P* < 0.01; †††, *P* < 0.001; ††††, *P* < 0.0001) and the Cas9-only control (*, *P* < 0.05; **, *P* < 0.01; ***, *P* < 0.001; ****, *P* < 0.0001) were made by Kruskal-Wallis analysis with Dunn’s posttest.

We assessed the impact of *PRA1* or *ZRT1* disruption on the fitness of *Blastomyces* in a mouse model of blastomycosis. Intratracheal injection of four out of five *pra1* mutant strains and one *zrt1* mutant strain resulted in a reduction of the fungal burden in the lungs by 1 to 3 orders of magnitude relative to that of the wild-type strain (Fig. 4). The Cas9-only control strains had a fungal load comparable to that of the wild-type *Blastomyces* 26199 strain. Combined disruption of *PRA1* and *ZRT1* did not further impair fitness beyond the reduction of the fungal burden observed in the single mutant strains, suggesting that these defects are not synergistic. This is consistent with observations in C. albicans, where Pra1 is a secreted zinc-scavenging protein that can interact with the cell surface high-affinity zinc transporter Zrt1 (4).

### Disruption of *PRA1* or *ZRT1* under zinc restriction leads to adaptation.

**FIG 5:**
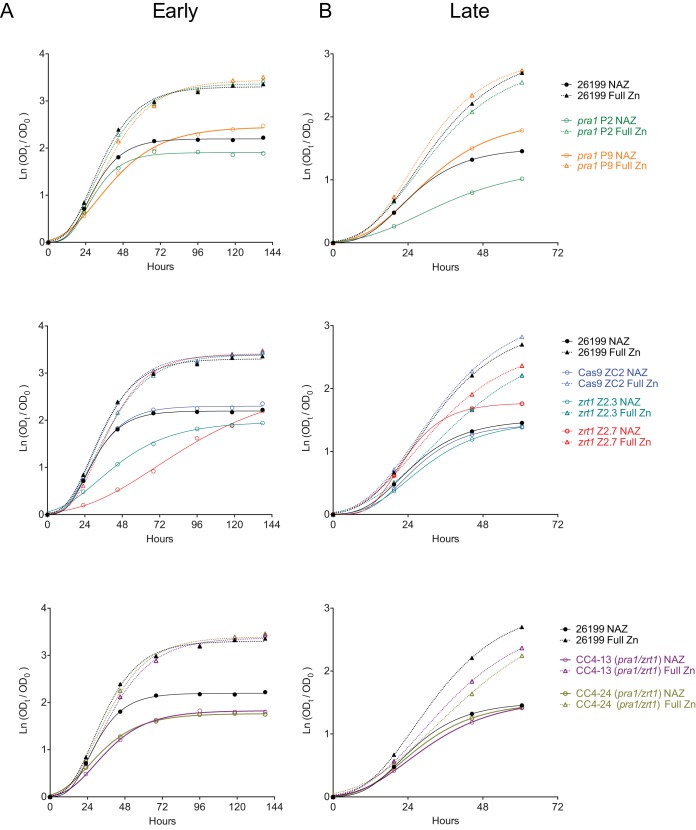
Growth of *pra1*, *zrt1*, and *pra1 zrt1* mutant *Blastomyces* cells under zinc-limited conditions shows adaptation. Growth in zinc-replete (Full Zn; dotted lines) or zinc-limited (No added Zn [NAZ]; solid lines) medium in early passage cycles (A) or after three additional passage cycles (B) within the same experiment is shown. Individual targeted strains are indicated on the right. CC4-13 and CC4-24 are *pra1 zrt1* double-mutant strains.

In many yeast species, intracellular zinc reserves are stored within the vacuole and are mobilized with vacuolar zinc transporters during times of low zinc availability (22, 23). It might therefore be expected that extended growth under zinc-limiting conditions could deplete intracellular stores and that this could potentially unmask more severe growth or fitness phenotypes in mutants with defects in the zinc acquisition machinery than would be observed under bountiful-zinc conditions. We therefore compared the growth of wild-type, Cas9 control, and *pra1* and/or *zrt1* mutant strains in low-zinc (no added zinc [NAZ]; approximately 0.5 μM) or standard-zinc (full zinc; approximately 3 μM) medium. All strains grew more slowly in low-zinc medium than under zinc-replete conditions, regardless of the genotype. Initially, the *zrt1* and *pra1 zrt1* mutants showed a defect in growth in low-zinc medium at day 3 versus wild-type and Cas9 controls (Fig. 5A). In later experiments performed after additional passage, however, the same mutant strains grew similarly to controls (Fig. 5B). This variable behavior could suggest that the mutant strains underwent adaptation to zinc limitation that involves additional genes besides *PRA1* and *ZRT1*.

We also examined whether prolonged zinc starvation (to deplete intracellular zinc stores) would exacerbate the reduction of fitness we observed previously in the *pra1* and *zrt1* mutant strains. Intratracheal injection of mutant and control strains after three serial passages in low-zinc medium led to a reduction in the fungal burden by a factor of approximately 5 to 105 in *pra1*, *zrt1*, and *pra1 zrt1* mutants relative to the wild-type strain (Fig. S3). This is within the same range of decreased fitness observed for these mutants grown under zinc-replete conditions (Fig. 4); thus, extended zinc limitation did not accentuate the fitness phenotype of these mutant strains.

10.1128/mBio.00412-18.3 FIG S3  Fitness testing after prolonged zinc starvation. Extended zinc starvation does not further reduce the fungal burden in *pra1* or *zrt1* mutant strains beyond that observed in cultures grown under zinc-replete conditions. Cultures were serially passaged for three cycles of growth in zinc-limited (No added Zn [NAZ]) medium for 6, 4, and 3 days, respectively, prior to harvest. Approximately 20,000 *Blastomyces* cells were administered per mouse, and lung homogenates were plated for CFU counting at 12 days postinfection. Comparisons of the total numbers of CFU per lung versus wild-type 26199 (†, *P* < 0.05; ††, *P* < 0.01; ††††, *P* < 0.0001) and versus the Cas9-only control (*, *P* < 0.05; **, *P* < 0.01; ****, *P* < 0.0001) were made by Kruskal-Wallis analysis with Dunn’s posttest. Sample sizes were 7 to 10 mice per group. Download FIG S3, EPS file, 1 MB. Copyright © 2018 Kujoth et al. 2018 Kujoth et al. This content is distributed under the terms of the Creative Commons Attribution 4.0 International license.

## DISCUSSION

In this work, we demonstrate that CRISPR/Cas9 technology can be applied to the thermally dimorphic fungus B. dermatitidis with high editing efficiencies comparable to those obtained with CRISPR/Cas9 systems and other model organisms. This represents a dramatic increase in editing efficiency over that of previously developed methods of gene disruption in *Blastomyces*. Additionally, we have demonstrated that dual-gene targeting can be achieved by the simple incorporation of a second sgRNA region and a short linker into the single-promoter–sgRNA–terminator expression cassette of the targeting vector. We expect that it will be possible to further increase the number of sgRNA regions within a single-promoter-driven cassette to expand the number of targets within a single experiment. The efficiency and flexibility of this system permit the simultaneous targeting of multiple isoforms within a gene family or of multiple genes with a biochemical, signaling, or regulatory pathway.

We utilized two strategies for dual-gene targeting—a single promoter driving the expression of both sgRNAs and separate promoters driving individual sgRNA cassettes. We obtained a much higher number of double mutants by using the single-promoter approach. Whether this is a reproducible advantage of the single-promoter system is not yet known, nor is the reason for this outcome. One possible explanation may be related to the recycling of the glyceraldehyde-3-phosphate dehydrogenase (*GAPDH*) promoter to drive each separate sgRNA cassette in the dual-promoter approach. In mammalian systems, multiple occurrences of the same promoter within a vector can result in suboptimal expression due to competition for the same transcription factors, a phenomenon referred to as “promoter interference” (24). If this is the case in our system, substitution of a different promoter for the second sgRNA cassette may be advantageous. Nevertheless, the success of the single-promoter strategy makes it the more attractive approach moving forward.

A consistent feature of CRISPR/Cas9 targeting in *Blastomyces* has been the relatively frequent observation of targeted clones bearing multiple mutations or containing a mixture of mutant and wild-type sequences. We interpret this as a reflection of heterokaryosis in multinucleate *Blastomyces* cells. The Cas9 protein in this system has been engineered to contain an exogenous nuclear localization signal (9) and therefore should be imported into all of the nuclei of a *Blastomyces* cell. In contrast, sgRNA transcript expression might be expected to occur locally only within an individual targeted nucleus, hence restricting successful targeting to that particular nucleus. Homokaryosis would then require segregation or purifying selection of edited nuclei during colony formation. The CRISPR/Cas9 targeting vectors are introduced into *Blastomyces* by *Agrobacterium*-mediated gene transfer. This method has been proven to reliably introduce foreign DNA into dimorphic fungi and produce homokaryons (6), however, so it is not clear why heterokaryons should be generated more prominently in the context of CRISPR/Cas9 editing. We do note that continued hygromycin B selection for up to eight passages after colony picking did not drive heterokaryotic clones to homokaryosis. Earlier work employing *Agrobacterium*-mediated transformation of *Blastomyces* cells (6) showed that homokaryotic clones developed even in the absence of antibiotic selection, and mathematical modeling suggested that under assumptions of random nuclear sorting, homokaryotic clones could occur within ~20 generations, although that number increases as the number of nuclei per cell increases (6). Despite the persistence of heterokaryotic transformants, we have obtained homokaryotic clones in all of our targeting experiments to date, and the high editing efficiencies of this approach suggest that the occurrence of heterokaryotic clones can be overcome by screening sufficient (but still easily practical) numbers of transformants.

In the absence of an exogenous repair template, double-strand breaks generated by the Cas9 nuclease are repaired by NHEJ, a nearly universal error-prone repair pathway that generates primarily small insertions and deletions (25–27). We found that to indeed be the case in *Blastomyces* as well, where single-base indels are the most frequent mutational events recovered, with a small number of 2- to 12-base indels also being observed. Interestingly, the specific bases inserted at the expected Cas9 cleavage site were heavily weighted by the protospacer sequence context (e.g., a G insertion was most prevalent in the *PRA1* protospacer, while A and C insertions were recovered in *ZRT1* protospacers 1 and 2, respectively). This pattern corresponds to the insertion of the identical base directly upstream of the cut site (i.e., the same base as the residue 4 bases upstream of the PAM). A large-scale analysis of repair outcomes following CRISPR/Cas9 editing in human cells similarly found that the distribution of specific indel mutations was nonrandom and dependent upon the protospacer sequence context (27).

The specificity of CRISPR/Cas9 gene editing has been investigated in numerous model systems (28–31), and the extent of off-target mutations has been debated (for examples, see references 32 and 33). We did not ascertain off-target editing activity in our system. Initial assessments of off-site targeting relied upon examination of the extent of mutations at genomic sites predicted to be potential targets based on one or a few mismatches within the protospacer sequence. This biased approach is of limited value because many characterized off-target mutations turn out to occur at locations that would not have been predicted by using such an approach (13, 34). Alternatively, unbiased methods of cataloging off-target mutations, such as GUIDE-Seq (34), follow the incorporation of a short oligonucleotide tag at all sites of double-strand breaks, which can then be identified by whole-genome sequencing. Such methods are not currently feasible in *Blastomyces* because of low electroporation efficiency and the resulting difficulty of introducing the marker oligonucleotides into the cell. With multiplexed CRISPR/Cas9 editing, however, it may be possible to include a tag sequence in the targeting vector that could be released by Cas9 cleavage directed by two sgRNAs targeted to vector sequences at both borders of the tag; this would be in addition to the sgRNA specific for the target protospacer of interest and so awaits a future demonstration of successful triplexed targeting. (Similarly, such an approach might also allow for the release of a DNA fragment that could be utilized as a homology-directed repair template for the introduction of specific sequence changes.) In the absence of direct monitoring of off-site mutations, we have used multiple clones of a targeted gene and multiple sgRNAs to a given target to reduce the likelihood that phenotypic consequences of random insertional or sgRNA-associated off-target mutational events will be misattributed to specific gene targeting. We did observe some phenotypic variation among mutants and cannot exclude the possibility that variation resulted from off-target activity or random integration.

Another common strategy is to use genetic complementation in CRISPR/Cas9-targeted organisms to rescue an observed phenotype and confidently assign a function to a particular gene of interest. In the current system, the expression of Cas9 and the sgRNAs is driven by constitutive promoters, and this is confirmed by our detection of Cas9 mRNA by qPCR in cells after multiple passages. This continued expression has implications for the use of genetic complementation to restore the function of targeted genes, as exogenous transgene copies would become templates for CRISPR/Cas9 editing unless silent mutations are incorporated into the protospacer region of the complementing gene sequence. The altered codon usage associated with silent mutations could potentially have effects on the level of expression from the transgene as well (35).

Zinc is the second most abundant metal in the human body and is critical for cell function, as it is a structural or catalytic cofactor for a myriad of proteins, such as transcription factors, RNA polymerase, oxidoreductases, hydrolases, ligases, and others, that affect transcription, macromolecule synthesis and degradation, and antioxidant defenses (e.g., Cu/Zn-superoxide dismutase), among many cellular processes (36). Thus, sequestration of zinc by vertebrate hosts is a key component of nutritional immunity to microbial pathogens. One key means of restricting zinc is through its high-affinity binding to calprotectin, a heterodimeric protein component of extracellular traps (NETs) released by neutrophils that have been stimulated to undergo netosis in response to fungal infections (37). Calprotectin has been shown to be critical for the antifungal activity of NETs, as anti-calprotectin antibodies and calprotectin deficiency in mice both impair activity (37). In the case of defense against intracellular fungal pathogens, increased zinc sequestration into the Golgi apparatus by ZnT class transporters and upregulation of zinc-binding metallothioneins have been observed in macrophages infected with another dimorphic fungus, Histoplasma capsulatum (38).

To survive and replicate within the host environment, pathogens must evolve strategies for overcoming such nutritional immunity to obtain zinc from host tissue compartments. In Saccharomyces cerevisiae, zinc import into the cell is mediated by high- and low-affinity zinc transporters Zrt1 and Zrt2, respectively, both of which are members of the ZIP family of ion transporters. Intracellular zinc is moved into the yeast vacuole by Zrc1 and Cot1 zinc transporters of the ZnT family and mobilized back into the cytosol by another ZIP protein, the vacuolar zinc transporter Zrt3. Regulation of the ZRT family members falls under the control of the transcription factor Zap1, which upregulates itself and *ZRT* genes under low-zinc conditions. Pathogenic fungi have analogous systems, which have been linked to virulence in these organisms. The human pathogen C. albicans has zinc transporters Zrt1 and Zrt2, as well as the *ZAP1* ortholog *CSR1* and the secreted zincophore Pra1. The Pra1 protein is required for efficient invasion of epithelial cells by fungal hyphae (4). A. fumigatus possesses multiple zinc transporters, including orthologs of *ScZRT1* (*zrfA*), *ScZRT2* (*zrfB*), and *CaZrt1* (*zrfC*), as well as two other predicted transporters (39); additionally, the *ZAP1* ortholog *zafA* and the *PRA1* ortholog *aspF2* are also present in A. fumigatus. Importantly, deletion of *zafA* or *zrfC* results in attenuation of virulence in a murine aspergillosis model (40, 41). Similarly, reduced virulence is observed in a mouse cryptococcosis model following deletion of the Cryptococcus gattii *ZAP1* transcription factor (42). Notably, single deletion of either *ZIP1*, which is similar to *zrfA* and *zrfB*/*CaZRT2*, or the *CaZRT1*/*zrfC* ortholog *ZIP2* has little effect on *Cryptococcus* virulence, whereas their combined loss dramatically diminishes virulence (43). In contrast, deletion of the *ScZRT1* ortholog *ZIP1* is sufficient to attenuate the virulence of inhaled Cryptococcus neoformans in mice (18). In H. capsulatum, RNA interference-mediated knockdown of *ZRT2* leads to a reduced fungal burden in mice (44). The impaired *Blastomyces* fitness observed upon the loss of *PRA1* and *ZRT1* adds to these findings to highlight the importance of zinc acquisition for the pathogenic potential of these multiple fungal species.

We expected that targeted disruption of the zinc acquisition genes would result in a reproducible *in vitro* growth disadvantage in zinc-limited medium. The initial reduction of growth we observed in the *zrt1* and *pra1 zrt1* mutant strains in early zinc restriction experiments was not observed in later trials, however. Additionally, extended growth in zinc-restricted medium did not further exacerbate the fitness impairment exhibited by the mutant strains. If zinc acquisition is important for fungal growth and survival, why was growth under low-zinc conditions so variable and why was fitness not more dramatically affected? Presumably, our results reflect an adaptation of the mutant strains to low-zinc conditions in culture. *Blastomyces* also contains a gene, *ZRT2*, that encodes a second cell surface zinc transporter, and it may be that it can provide some functional redundancy when *ZRT1* is lost, similarly to cryptococcal *ZIP1* and *ZIP2*. Another potential explanation is that the *PRA1 ZRT1* pathway is most important in the acquisition of zinc from tightly bound zinc stores rather than the more readily available “free” zinc in liquid culture medium (which might also be imported by Zrt2, for example). We tested this idea by growing *pra1*, *zrt1*, and *pra1 zrt1* mutants in full-zinc medium in the presence or absence of the zinc chelator *N*,*N*,*N*′,*N*′-tetrakis(2-pyridinylmethyl)-1,2-ethanediamine (TPEN); the mutant strains did not show any reproducible growth defect, however (data not shown). It is also possible that we failed to fully deplete intracellular zinc stores in our experiments. Mobilization of vacuolar zinc by Zrt3 in S. cerevisiae (23) can provide enough available zinc to sustain up to eight generations of cell division (22). We grew *pra1* and/or *zrt1* mutants through three successive growth curve and dilution cycles prior to injection into mice. On the basis of the doubling times we observed and the total time in the exponential growth phase through the duration of the experiment, this would correspond to approximately 11 or more generations in zinc-limited medium prior to *in vivo* fitness testing. Although zinc starvation prior to fitness testing *in vivo* did not exacerbate the reduction of the fungal burden of the mutant strains, *PRA1* and/or *ZRT1* disruption was nevertheless associated with impaired fitness across multiple experiments.

Our results have highlighted the contributions of zinc scavenging genes to the fungal pathogenesis of B. dermatitidis in an animal model of infection. Beyond this particular application, however, the adaptation of CRISPR/Cas9 technology to the dimorphic fungi should accelerate the pace at which specific gene function can be interrogated in these important but understudied human pathogens.

## MATERIALS AND METHODS

### *Blastomyces* strain generation.

B. dermatitidis strain 26199 was originally obtained from the American Type Culture Collection. B. dermatitidis strain 26199 Bad1-TRΔ20-Eα-*mCherry* T85-14.5 expressing an mCherry-tagged fusion protein was previously described (11). Cultures were grown at 37°C on oleic acid-albumin-dextrose-catalase-supplemented Middlebrook 7H10 (Becton, Dickinson Difco, Sparks, MD) slants or HMM plates (45).

### Plasmid vector construction.

The system used for CRISPR/Cas9-mediated *Blastomyces* targeting was adapted from that described by Nødvig et al. (9) for gene editing in filamentous fungi. The expression of the *cas9* gene from Streptococcus pyogenes, codon optimized for *Aspergillus* and containing a simian virus 40-derived nuclear localization signal, is controlled by the Aspergillus nidulans *tef1* promoter and terminator sequences. A hybrid sgRNA cassette consists of the sgRNA flanked by hammerhead and hepatic delta virus ribozymes, to allow autoexcision of the sgRNA from the hybrid transcript, which is expressed under the control of the A. nidulans *gpdA* promoter and *trpC* terminator. A *trpC* promoter (also from A. nidulans) drives the *hph* gene to allow for hygromycin B selection. These elements were placed into a binary vector (pPTS608) containing T-DNA border repeats to allow *Agrobacterium*-mediated *Blastomyces* transformation.

Plasmids pFC332 and pFC334 were kind gifts from Martin Kogle and Uffe Mortensen. A 7.25-kb fragment of pFC332 containing both the Ptef1-cas9-Ttef1 and PtrpC-hph cassettes was amplified with primers tdsP984 and tdsP985 (Table S2). This fragment was gel purified and assembled into the PacI- and PmeI-digested backbone of the binary vector pPTS608 (itself a derivative of pPZPTK8.10 [46]) with Gibson Assembly (47) Master Mix (New England Biolabs, Ipswich, MA) to create pPTS608-Cas9-hyg. The hybrid sgRNA cassettes specific for targeting genes of interest were created by PCR amplification of overlapping fragments A and B (Fig. S4) with primers containing the 20-nucleotide (nt) protospacer and 6-nt ribozyme inverted repeat overlapping the end of the protospacer (because the 5′ end of the hammerhead ribozyme base pairs with sequences at the start of the protospacer, each targeting vector must replace not only the 20-nt protospacer but also the 6 nt of the hammerhead inverted repeat needed to maintain complementarity to the protospacer sequence; Fig. 1A). The primers used to generate fragments A and B for all of the CRISPR/Cas9 targeting constructs are shown in Table S2. Fragments A and B were combined with PacI-digested pPTS608-Cas9-hyg to generate pPTS608-Cas9-hyg-target-sgRNA plasmids (where the target is *PRA1*, *ZRT1*, *BL-ENG2*, or *mCherry*) by Gibson assembly.

10.1128/mBio.00412-18.4 FIG S4  Detailed cloning diagram for CRISPR vector construction. (A) Replacement of gene-specific protospacers in the CRISPR/Cas9 targeting vectors is done by generating two overlapping fragments, A and B, that create the entire promoter-sgRNA-terminator cassette, which is then inserted into the vector backbone (not shown, but see Fig. 1B). Gene-specific sequences are included in the overlapping inner primers (tdsP987 and tdsP988 in this case). (B) A detailed view of the overlapping inner primer region illustrates that the 20-nt gene-specific protospacer sequence is included in the fragment B forward primer (e.g., tdsP988). Because the 5′ end of the hammerhead ribozyme forms an inverted repeat with the first six bases of the protospacer (HH IR′), the corresponding six bases in the 5′ hammerhead region must also be replaced with a gene-specific sequence (blue box) in the fragment A reverse primer. HH, hammerhead ribozyme sequence; HDV, hepatitis delta virus ribozyme sequence; PgpdA, A. nidulans *gpdA* promoter; TtrpC, A. nidulans *trpC* terminator; IR, inverted repeat forming region of the HH ribozyme; tdsP986-tdsP989, primers. Download FIG S4, EPS file, 2.2 MB. Copyright © 2018 Kujoth et al. 2018 Kujoth et al. This content is distributed under the terms of the Creative Commons Attribution 4.0 International license.

10.1128/mBio.00412-18.6 TABLE S2  Primers used for plasmid construction and qPCRs in this study. Download TABLE S2, PDF file, 0.1 MB. Copyright © 2018 Kujoth et al. 2018 Kujoth et al. This content is distributed under the terms of the Creative Commons Attribution 4.0 International license.

To construct the dual-promoter, dual-targeting vector (Fig. 1B, diagram iii), the PgpdA-*ZRT1* sgRNA no. 1-TtrpC fragment from pPTS608-Cas9-hyg-*ZRT1* no. 1 was amplified by PCR with primers gckP034 and gckP035 (Table S2) and the resulting ~1-kb product was gel purified and inserted by Gibson assembly into pPTS608-Cas9-hyg-*PRA1* that had been linearized by digestion with BglII, which cuts upstream of the *PRA1* sgRNA expression cassette. This produced pPTS608-Cas9-hyg-2X-*PRA1*-*ZRT1*.

The single-promoter, dual-targeting vector pPTS608-Cas9-hyg-2XSP-*PRA1*-*ZRT1* was constructed by Gibson assembly of three fragments, (i) a PacI-linearized pPTS608-Cas9-hyg backbone, (ii) a PgpdA-*PRA1* sgRNA PCR fragment amplified with primers gckP048 and gckP049, and (iii) a *ZRT1* no. 1 sgRNA-TtrpC PCR fragment amplified with primers gckP050 and gckP053. Primers gckP049 and gckP050 also contained complementary sequences to create a random 15-bp linker (CTTGTACATTGAGAG) between the ribozyme-flanked *PRA1* and *ZRT1* sgRNAs (Fig. 1B, diagram iii).

All cloning PCRs were done with Q5 high-fidelity polymerase (New England Biolabs, Ipswich, MA), and the sgRNA/protospacer regions of all targeting constructs were confirmed by Sanger DNA sequencing.

Candidate protospacer sequences in the targeted genes were generated by CHOPCHOP (48, 49), and high-scoring candidates near the N-terminal region of the protein coding sequence were searched against the B. dermatitidis genome by using BLAST to eliminate candidates with obvious potential off-target sites.

### *Agrobacterium*-mediated *Blastomyces* transformation.

The use of *Agrobacterium*-mediated *Blastomyces* transformation was developed previously (6). Agrobacterium tumefaciens strain LBA1100 carrying Ti plasmid pAL1100 (50) was electroporated with 20 ng of targeting vector (2.5 kV, 25 μF, 200 Ω) and grown at 28°C for 3 days on LB plates containing 0.1% glucose, kanamycin (100 μg/ml), and spectinomycin (100 μg/ml, to select for maintenance of the Ti plasmid). Two days prior to cocultivation, transformants were seeded into 5 ml of *Agrobacterium* minimal medium (51) containing kanamycin and spectinomycin (100 μg/ml each) and grown overnight with shaking at 28°C. The culture was diluted to an *A*_600_ of 0.05 in induction medium (52) supplemented with 200 μM acetosyringone and grown overnight at 28°C. The cultures were harvested at an *A*_600_ of ~0.6, and 100 µl of *Agrobacterium* was mixed with an equal volume of B. dermatitidis 26199 yeast (~1 × 10^7^ cells) that had been harvested in liquid HMM after 2 to 4 days of growth. The cocultivation mixtures were spread on Biodyne A nylon membranes (Pall Gelman, Ann Arbor, MI) placed on induction medium plates containing 200 μM acetosyringone. After 2 to 3 days of growth at 28°C, the membranes were switched to 3M plates containing 200 μM cefotaxime (Sigma) and 25 μg/ml hygromycin B (A.G. Scientific, San Diego, CA) and grown under selection for 2 to 3 weeks at 28°C. Colonies of hygromycin B-resistant *Blastomyces* transformants were expanded under selection for at least two passages prior to growth on 7H10 slants or HMM plates for routine maintenance and experimental use.

### Screening of targeted clones.

*Blastomyces* transformants were screened by PCR and sequencing. *Blastomyces* genomic DNA was isolated with the MasterPure yeast DNA purification kit (Epicentre Technologies, Madison, WI), extending the 65°C incubation to at least 1 h. Genomic DNA regions surrounding the protospacer region in the target gene of interest were amplified by PCR with Phusion DNA polymerase (New England Biolabs) and primers (Table S2) gckP001 and gckP002 (*PRA1*), gckP062 and gckP064 (*ZRT1*), gckP065 and gckP068 (*mCherry*), and gckP042 and gckP043 (*BL-ENG2*). PCR products were cleaned up with the QIAquick PCR purification kit (Qiagen) or gel purification prior to Sanger sequencing with the BigDye Terminator v3.1 cycle sequencing kit (Amersham) and the same primers used for PCR. Sequence reads were aligned with the corresponding reference sequences with MacVector 15.5.3.

### RNA preparation, cDNA synthesis, and RT-qPCR.

Wild-type or *pra1 zrt1* mutant *Blastomyces* clones were inoculated into either zinc-replete or zinc-limited liquid HMM (see growth curve determination below) and grown for >2 days prior to dilution passage to a target optical density at 600 nm (OD_600_) of 0.8 to 1.0. After overnight growth, cells were harvested by centrifugation for 10 min at 800 to 1,200 × *g*, washed in cold 1× phosphate-buffered saline (PBS), and frozen drop by drop in liquid nitrogen. Frozen fungal pellets were ground into a fine powder with a liquid nitrogen-cooled mortar and pestle and dissolved in 10 ml of RNAzol RT (Molecular Research Center, Cincinnati, OH), and DNA, protein, and polysaccharides were precipitated by the addition of 4 ml of diethyl pyrocarbonate (DEPC)-treated water and vigorous mixing. Following incubation for 15 min at room temperature, samples were centrifuged at 4°C for 15 min at 12,000 × *g*. The aqueous phase was transferred to a new tube, and the RNA was precipitated by 10 min of incubation with 4 ml of 75% ethanol, followed by centrifugation at 4°C for 8 min at 12,000 × *g*. The RNA pellet was washed with 70% ethanol and resuspended in DEPC-treated water, and the concentration was measured by NanoDrop spectrophotometry. RNA preparations were treated with 6 U of Turbo DNase (Ambion, Austin, TX) for 30 min at 37°C and processed with a Qiagen RNeasy Mini cleanup kit in accordance with the manufacturer’s instructions. RNA was eluted in RNase-free water, and the concentration was measured by NanoDrop spectrophotometry.

Up to 1 µg of DNase-treated, RNeasy-cleaned RNA was used as the template in each iScript cDNA synthesis kit reaction (Bio-Rad, Hercules, CA) in accordance with the manufacturer’s instructions. Equivalent RNA input amounts were used for all of the samples being compared in each experiment. Control synthesis reaction mixtures lacking the reverse transcriptase enzyme were also prepared to monitor amplification of carryover genomic DNA. The cDNA products were diluted 1:10 prior to use in qPCRs.

RT-qPCRs were performed in accordance with the manufacturer’s instructions with SsoFast EvaGreen Supermix (Bio-Rad), the primers indicated in Table S2, and equivalent cDNA product volumes for all samples. Real-time detection and analysis of amplicon fluorescence were performed with a Rotor-Gene Q thermocycler (Qiagen) and its associated Rotor-Gene Software, version 2.3.1. *PRA1*, *ZRT1*, or *Cas9* targets were normalized to *GAPDH* or ribosomal protein large subunit 34 (*RPL34*) reference gene expression and plotted relative to 26199 full-zinc samples. All samples were assayed in triplicate in each experiment. Normalization of target gene expression and fold change calculation were performed by the 2^−ΔΔ*CT*^ method of Livak and Schmittgen (53). Relative expression ratios were calculated with respect to the wild-type zinc-replete control.

### Western blotting.

Secreted protein samples used in Western blot analyses were obtained from the same cultures used to generate RNA preparations (see above). At the time of harvesting for RNA, conditioned medium from wild-type or targeted *Blastomyces* cultures grown in either zinc-replete or zinc-limited liquid HMM was collected as the supernatant from harvest centrifugation for 10 min at 800 to 1,200 × *g*. Supernatants were stored frozen at −70°C. Subsequently, thawed supernatant was concentrated ~20× with Centriprep Ultracel YM-3 spin columns with a 3,000 molecular weight cutoff (Millipore) at 2,000 × *g* for 4 h, and protein concentrations were determined by bicinchoninic acid assay (Pierce). Protein concentrations were equalized prior to loading onto a 10% polyacrylamide gel, and electrophoresis proceeded for 120 min at 100 V. Proteins were transferred onto polyvinylidene difluoride membranes with the Trans-Blot Turbo system (Bio-Rad). Membranes were blocked in PBS plus 1% bovine serum albumin at 4°C for 1 h prior to incubation with rabbit anti-Pra1 polyclonal antibody (20, 21) (a kind gift from Peter Zipfel) for 2 h at room temperature. Membranes were washed with PBS plus 0.1% Tween 20 three times for 10 min each and probed with a horseradish peroxidase-conjugated goat anti-rabbit secondary antibody. After washing in accordance with the Clarity Western ECL kit (Bio-Rad) instructions and permeation with substrate, bands were visualized with a VersaDoc 5000 imaging system (Bio-Rad).

### Growth curve determination.

Wild-type and targeted *Blastomyces* strains were carried in liquid HMM for one or two passages prior to the start of growth curve determination to allow adaptation from maintenance 7H10 slants or HMM plates. Growth curve cultures were inoculated at an OD_600_ of 0.1, and daily OD_600_ measurements were recorded. For determination of lag times, doubling times, and maximum growth, the natural log of the ratio of the observed OD_600_ at time *t* to the initial OD was plotted versus time, and a sigmoidal growth curve was modeled by fitting to the Gompertz equation as modified by Zwietering (54). The slope of the tangent at the inflection point gives the maximal growth rate (μ), and the asymptote of the plateau phase represents maximal growth. Doubling times were calculated as the natural log of 2 divided by μ. The lag time corresponds to the *x* intercept of the tangent at the inflection point.

Initial assessments of growth rates were done in standard liquid HMM (3 μM zinc). For experiments requiring growth under zinc-limited versus zinc-replete conditions (including growth curves, RNA expression, and Western blotting), low-zinc HMM and zinc-reconstituted HMM were prepared as follows. All medium components excluding Ham’s F-12 (the major source of metals) were mixed and metals were stripped in batches with Chelex 100 resin (10%, wt/vol; Bio-Rad). Custom Ham’s F-12 formulated without zinc (GE Healthcare Life Sciences) was then added prior to filter sterilization. Batches of medium were blended to ensure uniform composition. Residual zinc content was quantified with a colorimetric 4-(2-pyridylazo)resorcinol (PAR) assay (55). Zinc was then reconstituted to a target of 3 μM for zinc-replete medium with zinc sulfate, whereas no zinc reconstitution was done for zinc-limited medium. Final zinc content was verified by PAR assay and registered at 0.3 to 0.6 and 2.7 to 3.3 μM for zinc-limited and zinc-replete media, respectively. Medium preparation and storage were done with virgin plasticware, and cultures were grown in acid-washed glassware.

### Fungal burden testing.

*Blastomyces* cells were harvested from HMM plates with PBS, passed through a 40-μm nylon mesh cell strainer (Fisher Scientific), and counted on a hemocytometer. Cells were resuspended in PBS at a density of 10^6^/ml, and 20,000 cells (20 μl) were injected intratracheally into male C57BL6/NCr mice (National Cancer Institute via Charles Rivers Laboratories). Lungs were harvested at 10 to 15 days postinfection and placed into 2 ml of PBS for homogenization with a motorized tissue grinder (Omni International, Kennesaw, GA). Lung homogenates were diluted by a factor of 20 to 2 × 10^5^ and plated onto BBL brain heart infusion (Becton, Dickinson Difco, Sparks, MD) agar plates. Colonies were counted 4 to 8 days later, and the total numbers of CFU per lung were displayed as box-and-whisker plots. In each experiment, 10 mice per strain were injected; typically, several mice in an experiment died from complications of the surgery or succumbed to *Blastomyces* infection prior to euthanasia. In no case did the sample size fall below 6 mice per strain, and the majority of strains (90%) had 8 to 10 mice per group per experiment.

### Statistical analysis.

All statistical analyses were performed with Prism 6 software (GraphPad, La Jolla, CA). Comparisons of growth parameters and CFU burdens were assessed by Kruskal-Wallis analysis with Dunn’s posttest to correct for multiple comparisons. The family-wide significance level was set to *P* < 0.05.
