# Supplementary material for: CRISPR/Cas9-Mediated Gene Disruption Reveals the Importance of Zinc Metabolism for Fitness of the Dimorphic Fungal Pathogen Blastomyces dermatitidis
Source: mBio. 2018 Apr 3;9(2):e00412-18. doi: 10.1128/mBio.00412-18 (PMC5885028; doi:10.1128/mBio.00412-18)
Supplement: TABLE S1 [file mbo002183801st1.docx]

| **Table S1: *Blastomyces dermatitidis* mutant strains used for phenotyping** | | | |
| --- | --- | --- | --- |
| Strain | *PRA1* Protospacer | *ZRT1*  Protospacer #1 | *ZRT1*  Protospacer #2 |
| *PRA1* targeting: |  |  |  |
| P2 | ins G | n/d | n/d |
| P7 | ins G | n/d | n/d |
| P9 | ins G | n/d | n/d |
| P13 | ins G | n/d | n/d |
| P21b | del G | n/d | n/d |
|  |  |  |  |
| Cas9-only controls: |  |  |  |
| C2 | WT | n/d | n/d |
| ZC2 | n/d | WT | WT |
|  |  |  |  |
| *ZRT1* protospacer #1 targeting: | |  |  |
| Z1.5 | n/d | ins A | WT |
|  |  |  |  |
| *ZRT1* protospacer #2 targeting: | |  |  |
| Z2.3 | n/d | WT | ins C |
| Z2.7 | n/d | WT | ins C |
|  |  |  |  |
| 2XSP *PRA1 ZRT1* #1 targeting: | |  |  |
| CC4-10 | ins GCCAT | ins A | WT |
| CC4-13 | ins T | ins A | WT |
| CC4-24 | del GC | ins A | WT |
|  |  |  |  |

n/d, not determined
