# Supplementary material for: CRISPR/Cas9-Mediated Gene Disruption Reveals the Importance of Zinc Metabolism for Fitness of the Dimorphic Fungal Pathogen Blastomyces dermatitidis
Source: mBio. 2018 Apr 3;9(2):e00412-18. doi: 10.1128/mBio.00412-18 (PMC5885028; doi:10.1128/mBio.00412-18)
Supplement: TABLE S2 [file mbo002183801st2.pdf]

Table S2: Primers used for plasmid construction and qPCR

Primer name	Sequence (5' - 3')[1]	Target[2]	Purpose[3]
tdsP984	gtcaaacactgatagtttCTTGGTGATGCTAAAGGG	pFC332	GA: pPTS608-Cas9-hyg; amplify Cas9 & hyg backbone
tdsP985	attgtggtgtaaacattaatGTGTCGCCCTTATTCGAC	pFC332	GA: pPTS608-Cas9-hyg; amplify Cas9 & hyg backbone
tdsP986, FragA F (common)	ccgctgagggtttaatGCGTAAGCTCCCTAATTG	pFC334 or pPTS608-Cas9 sgRNA vectors	GA: CRISPR targeting constructs
tdsP989, FragB R (common)	cggctgaggtcttaatGAGCCAAGAGCGGATTCC	pFC334 or pPTS608-Cas9 sgRNA vectors	GA: CRISPR targeting constructs
tdsP991, FragA R (Pra1)	GACGAGCTTACTCGTTTCGTCCTCACGGACTCATCAGGCTGGGCGGTGATGTCTGCTCAAG	pFC334 or pPTS608-Cas9 sgRNA vectors	GA: pPTS608-Cas9-hyg-Pra1 sgRNA
tdsP992, FragB F (Pra1)	GAAACGAGTAAGCTCGTCGCTGGGTTCCAATCTGCATGGTTTTAGAGCTAGAAATAGC	pFC334 or pPTS608-Cas9 sgRNA vectors	GA: pPTS608-Cas9-hyg-Pra1 sgRNA
gckP001, PRA1 F	TCACCATGAGGCTCCCCATA	*B.d. PRA1* (BDFG 05357)	Screening & sequencing targeted strains
gckP002, PRA1 R	GCCATACCAGAGCACATGGT	*B.d. PRA1* (BDFG 05357)	Screening & sequencing targeted strains
gckP011, PRA1 F	GACTATCCCATCCACAAC	*B.d. PRA1* (BDFG 05357)	qPCR
gckP012, PRA1 R	TACAGAGCGGAATCTTTG	*B.d. PRA1* (BDFG 05357)	qPCR
gckP015, Cas9 F	GAGCATGCCGCAAGTCAATA	Cas9 (pPTS608-Cas9-hyg)	qPCR
gckP016, Cas9 R	CCCAATCCTTCTTTCGAGCG	Cas9 (pPTS608-Cas9-hyg)	qPCR
gckP021, GAPDH F	ACCCCCGCTCCTCCATCTTC	*B.g. GAPDH* (BDBG 07959)	qPCR
gckP022, GAPDH R	GAGTAGCCCCACTCGTTGTCATACC	*B.g. GAPDH* (BDBG 07959)	qPCR
gckP025, RPL34 F	AAAGTCCGCATCATCAAGAC	*B.g. RPL34* (BDBG 01400)	qPCR
gckP026, RPL34 R	GAACGCCTGGGAGTTTG	*B.g. RPL34* (BDBG 01400)	qPCR
gckP027, FragA R (Zrt1 #1)	GACGAGCTTACTCGTTTCGTCCTCACGGACTCATCAGATAGCACGGTGATGTCTGCTCAAG	pFC334 or pPTS608-Cas9 sgRNA vectors	GA: pPTS608-Cas9-neo-Zrt1.1 sgRNA
gckP028, FragB F (Zrt1 #1)	GAAACGAGTAAGCTCGTCATAGCAAGGCAATACGTAGGGTTTTAGAGCTAGAAATAGC	pFC334 or pPTS608-Cas9 sgRNA vectors	GA: pPTS608-Cas9-neo-Zrt1.1 sgRNA
gckP029, FragA R (Zrt1 #2)	GACGAGCTTACTCGTTTCGTCCTCACGGACTCATCAGCATGGTCGGTGATGTCTGCTCAAG	pFC334 or pPTS608-Cas9 sgRNA vectors	GA: pPTS608-Cas9-neo-Zrt1.2 sgRNA
gckP030, FragB F (Zrt1 #2	GAAACGAGTAAGCTCGTCCATGGTTATGGCAGTCGGAGGTTTTAGAGCTAGAAATAGC	pFC334 or pPTS608-Cas9 sgRNA vectors	GA: pPTS608-Cas9-neo-Zrt1.2 sgRNA
gckP034, F	gcggaacatatactgggcccgggaagatctGCGTAAGCTCCCTAATTGG	pPTS608-Cas9-hyg-Zrt1 sgRNA #1	GA: pPTS608-Cas9-hyg-2X-Pra1-Zrt1 #1 sgRNAs
gckP035, R	tcagcggaaacagctatgaccatgagatctGAGCCAAGAGCGGATTCC	pPTS608-Cas9-hyg-Zrt1 sgRNA #1	GA: pPTS608-Cas9-hyg-2X-Pra1-Zrt1 #1 sgRNAs
gckP042, F	ACAATCAGGCCGTGTTTGTA	*B.d. BLENG2* (BDFG 08749)	Screening & sequencing targeted strains
gckP043, R	ATTCCTTCTGCGCATCCTTT	*B.d. BLENG2* (BDFG 08749)	Screening & sequencing targeted strains
gckP044, FragA R (mCherry v1, v2)	GACGAGCTTACTCGTTTCGTCCTCACGGACTCATCAGGCGCTTCGGTGATGTCTGCTCAAG	pFC334 or pPTS608-Cas9 sgRNA vectors	GA: pPTS608-Cas9-hyg-mCherry sgRNA
gckP045, FragB F (mCherry v1)	GAAACGAGTAAGCTCGTCGCGCTTCAAGGTGCGCATGGGTTTTAGAGCTAGAAATAGC	pFC334 or pPTS608-Cas9 sgRNA vectors	GA: pPTS608-Cas9-hyg-mCherry sgRNA
gckP048, PgpdA-Pra1_sgRNA F	tagctgtttccgctgagggtttaattaaGCGTAAGCTCCCTAATTGGC	pPTS608-Cas9-hyg-Pra1 sgRNA	GA: pPTS608-Cas9-hyg-2XSP-Pra1-Zrt1 #1 sgRNAs
gckP049, PgpdA-Pra1_sgRNA R	gcactctcaatgtacaagGTCCCATTCGCCATGCCG	pPTS608-Cas9-hyg-Pra1 sgRNA	GA: pPTS608-Cas9-hyg-2XSP-Pra1-Zrt1 #1 sgRNAs
gckP050, Zrt1.1_sgRNA F	tggcgaatgggaccttgtacattgagagTGCTATCTGATGAGTCCGTGAG	pPTS608-Cas9-hyg-Zrt1 sgRNA #1	GA: pPTS608-Cas9-hyg-2XSP-Pra1-Zrt1 #1 sgRNAs
gckP053, TtrpC R	attctgctgtctcggctgaggtcttaattaaGAGCCAAGAGCGGATTCC	pPTS608-Cas9-hyg-Zrt1 sgRNA #1	GA: pPTS608-Cas9-hyg-2XSP-Pra1-Zrt1 #1 sgRNAs
gckP062, ZRT1 F	TATACCTCTATCCAAGTCCAACTA	*B.d. ZRT1* (BDFG 09159)	Screening & sequencing targeted strains
gckP064, ZRT1 R	CTCCTATGCAAAATCTAGATGGAA	*B.d. ZRT1* (BDFG 09159)	Screening & sequencing targeted strains
gckP065, mCherry F	GGAATAAGTGGTGCAACAAGC	Bad1 TRΔ20 Eα mCherry T85-14.5 gDNA	Screening & sequencing targeted strains
gckP068, mCherry R	CAGCTTCACCTTGTAGATGAACTC	Bad1 TRΔ20 Eα mCherry T85-14.5 gDNA	Screening & sequencing targeted strains

Primer name	Sequence (5' - 3')[1]	Target[2]	Purpose[3]
gckP069, FragB F (mCherry v2)	GAAACGAGTAAGCTCGTCGCGCTTCAAGGTGCACATGGGTTTTAGAGCTAGAAATAGC	pFC334 or pPTS608-Cas9 sgRNA vectors	GA: pPTS608-Cas9-hyg-mCherry sgRNA #2
gckP070, FragA R (mCherry v3)	GACGAGCTTACTCGTTTCGTCCTCACGGACTCATCAGGCGCATCGGTGATGTCTGCTCAAG	pFC334 or pPTS608-Cas9 sgRNA vectors	GA: pPTS608-Cas9-hyg-mCherry sgRNA #3
gckP071, FragB F (mCherry v3)	GAAACGAGTAAGCTCGTCGCGCATGAACTCCTTGATGAGTTTTAGAGCTAGAAATAGC	pFC334 or pPTS608-Cas9 sgRNA vectors	GA: pPTS608-Cas9-hyg-mCherry sgRNA #3
P1 FragA R (Bl-Eng2 #1)	GACGAGCTTACTCGTTTCGTCCTCACGGACTCATCAGAGTACTCGGTGATGTCTGCTCAAG	pFC334 or pPTS608-Cas9 sgRNA vectors	GA: pPTS608-Cas9-hyg-BlEng2 sgRNA #1
P2 FragB F (Bl-Eng2 #1)	GAAACGAGTAAGCTCGTCAGTACTCAGCTTGGCTAGAGGTTTTAGAGCTAGAAATAGC	pFC334 or pPTS608-Cas9 sgRNA vectors	GA: pPTS608-Cas9-hyg-BlEng2 sgRNA #1
P3 FragA R (Bl-Eng2 #2)	GACGAGCTTACTCGTTTCGTCCTCACGGACTCATCAGGAAGTCCGGTGATGTCTGCTCAAG	pFC334 or pPTS608-Cas9 sgRNA vectors	GA: pPTS608-Cas9-hyg-BlEng2 sgRNA #2
P4 FragB F (Bl-Eng2 #2)	GAAACGAGTAAGCTCGTCGAAGTCGTCAAAGAAGTTCGGTTTTAGAGCTAGAAATAGC	pFC334 or pPTS608-Cas9 sgRNA vectors	GA: pPTS608-Cas9-hyg-BlEng2 sgRNA #2

[1]Color coding for Gibson Assembly Frag A & B primers: Hammerhead (HH) ribozyme, HH IR or IR' in protospacer, protospacer, sgRNA backbone, gpd promoter, trpC terminator

[2]*B.g., Blastomyces gilchristii; B.d., Blastomyces dermatitidis*

[3]GA: Gibson Assembly
